# Placental T2* as a measure of placental function across field strength from 0.55T to 3T

**DOI:** 10.1038/s41598-024-77406-6

**Published:** 2024-11-19

**Authors:** Megan Hall, Jordina Aviles Verdera, Daniel Cromb, Sara Neves Silva, Mary Rutherford, Serena J. Counsell, Joseph V Hajnal, Lisa Story, Jana Hutter

**Affiliations:** 1 Department of Early Life Imaging, King’s College London, London, UK; 2 Centre for Medical Engineering, King’s College London, London, UK; 3 Department of Women’s and Children’s Health, King’s College London, London, UK; 4 Smart Imaging Lab, Radiological Institute, University Hospital Erlangen, FAU Erlangen, Bavaria, Germany; 5 St Thomas’ Hospital, 1st Floor, South Wing Westminster Bridge Road, SE1 7EH London, UK

**Keywords:** Placental MRI, T2* relaxometry, Fetal imaging, Biomedical engineering, Translational research, Prognostic markers

## Abstract

Placental MRI is increasingly implemented in clinical obstetrics and research. Functional imaging, especially T2*, has been shown to vary across gestation and in pathology. Translation into the clinical arena has been slow because of time taken to mask the region of interest and owing to differences in T2* results depending on field strength. This paper contributes methodology to remove these barriers by utilising data from 0.55, 1.5 and 3T MRI to provide a fully automated segmentation tool; determining field strength dependency of placental assessment techniques; and deriving normal ranges for T2* by gestational age but independent of field strength. T2* datasets were acquired across field strengths. Automatic quantification including fully automatic masking was achieved and tested in 270 datasets across fields. Normal curves for quantitative placental mean T2*, volume and other derived measurements were obtained in 273 fetal MRI scans and z-scores calculated. The fully automatic segmentation achieved excellent quantification results (Dice scores of 0.807 at 3T, 0.796 at 1.5T and 0.815 at 0.55T.). Similar changes were seen between placental T2* and gestational age across all three field strengths (*p* < 0.05). Z-scores were generated. This study provides confidence in the translatability of T2* trends across field strengths in fetal imaging.

## Introduction

Fetal MRI has been utilised for acquisition of anatomical data for over 30 years. However, advances in MRI, particularly functional imaging, have increased the clinical potential of MRI beyond characterisation of structural anomalies. In particular, this has proven valuable in improving our understanding of the human placenta, an organ for which there were previously very limited methods for in vivo study. The application of T2*-relaxometry has offered important insights into both physiological placental development across gestation^1–3^, as well as abnormal placentation as seen in pregnancies affected by hypertensive diseases^2,4,5^, prior to spontaneous preterm birth^6^, fetal growth restriction and discordant growth in twins^7,8^ albeit in small cohorts. A first multi-center study was recently published^1^. In addition to quantitative baseline T2*, multiple studies evaluating the effect of maternal hyperoxygenation on placental BOLD or T2* have been presented^9,10^.

Most studies evaluate mean T2* over the entire organ with some variation in terms of coverage, from 3 to 5 slices at predefined locations^3^ to whole organ coverage^11–13^. Other derived quantities have been suggested such as histogram based measures^14^ and texture-based scores were employed on T2-weighted imaging^11–13^. Visual assessment of the placenta, in particular, increased heterogeneity both over gestational age and in disease^15^ has also been described as an additional measure to mean T2*. Despite these advances, translation into the clinical setting has two major barriers: improved automation of output and translation of results across field strengths.

Quantitative analysis of the placenta requires accurate placental segmentation. However, it is a time-consuming task, highly reliant on the user’s expertise and affected by fetal and maternal motion, contractions and image artifacts. Automatic medical image segmentation helps establish standardised protocols across different users and centres, increasing accuracy, efficiency and scalability. Several studies have explored semi-automatic and automatic placenta segmentation in structural MRI acquisitions^16–21^. However, for quantitative techniques such as T2* acquisitions, the lower in-plane resolution and the poorer contrast between adjacent tissues increases complexity. Recent work by Abulnaga et al.^16^ and Pietsch^17^ et al. explore the automatisation of placental segmentation in BOLD images using 2D and 3D U-Net architectures and open a new path towards automatic post-processing. They were, however, based on data from one field-strength only and are in the latter case limited to mean T2* assessment.

While most clinical scanners operate at 1.5T, 3T scanners are often utilised in the research setting, due to the improved signal to noise ratio at higher field strengths. More recently there has been interest in the lower 0.55T field-strength for its potential to reduce B1 inhomogeneity, larger bore size, and potential to be more accessible as a consequence of reduced infrastructure requirements. While each field strength has unique advantages and so research has been undertaken at all three field strengths, T2* values are field strength dependent and there is currently no way of standardising results across field strengths.

This paper aims to contribute methodology to remove these barriers to clinical translation by utilising data from 0.55, 1.5 and 3T MRI of the placenta in vivo to: (1) evaluate field strength dependency across a range of placental assessment techniques; (2) determine normal ranges for T2* by gestational age but independent of field strength for use in future research studies and clinical obstetrics; and (3) provide a tool for fully automatic placental segmentation on gradient echo data from all field strengths from 0.55-3T.

## Methods

Women were recruited across seven studies at St. Thomas’ Hospital. Field strength used was dependent on study. Recruitment to this analysis was retrospective and based on a low risk pregnancy and normal maternal and neonatal outcomes for the morphological and quantitative analysis. Both healthy and pathological pregnancies were included for automatic placenta segmentation to ensure generalisability. Exclusion criteria to all studies included multiple pregnancies, claustrophobia or implants contraindication MRI, and maternal age under 16 years. All women provided informed, written consent prior to participation. In all scans, continuous heart rate and saturation monitoring was undertaken as well as intermittent blood pressure monitoring. For 3T, scanning was performed on a Philips Achieva scanner (bore size 60 cm) with a 32-channel cardiac coil, for 1.5T on a Philips Ingenia scanner (bore size 70 cm) with a 24 channel torso coil, and for 0.55T on a 0.55T Siemens Free.Max scanner (bore size 80 cm) with a 9-channel cardiac and a 6-channel posterior coil. Only scans acquired with the mother in supine position were included to maintain consistency^22^, as maternal position can affect placental perfusion^9,23^, with differences in T2* signal seen depending on whether the mother is supine or slightly tilted (left-lateral)^24^.

### Ethics

Scans were undertaken as part of the following studies: at 3T: Antenatal assessment of fetal infection using advanced MRI protocols (South East Scotland Ethics Committee, 19/SS/0032); Individual risk prediction of of adverse outcomes of pregnancies that deliver preterm using advanced MRI techniques and machine learning (South East Scotland Ethics Committee, 21/SS/0082); and Congenital Heart disease Imaging Programme (Wales Research Ethics Committee, 21/WA/0075). At 1.5T iFind (London, Dulwich, Research Ethics Committee14/L0/1806); The Placental Imaging Project (London, Dulwich, Research Ethics Committee 16/LO/1573); Cardiac and placental imaging in pregnancy (London, Dulwich, Research Ethics Committee 18/LO/1958); At 0.55T MRI examination enhanced by realtime knowledge during the acquisition time (London, Dulwich, Research Ethics Committee 19/LO/0852). All methodology followed relevant protocols, guidelines and regulations.

## Acquisition protocols

**Fig. 1:**
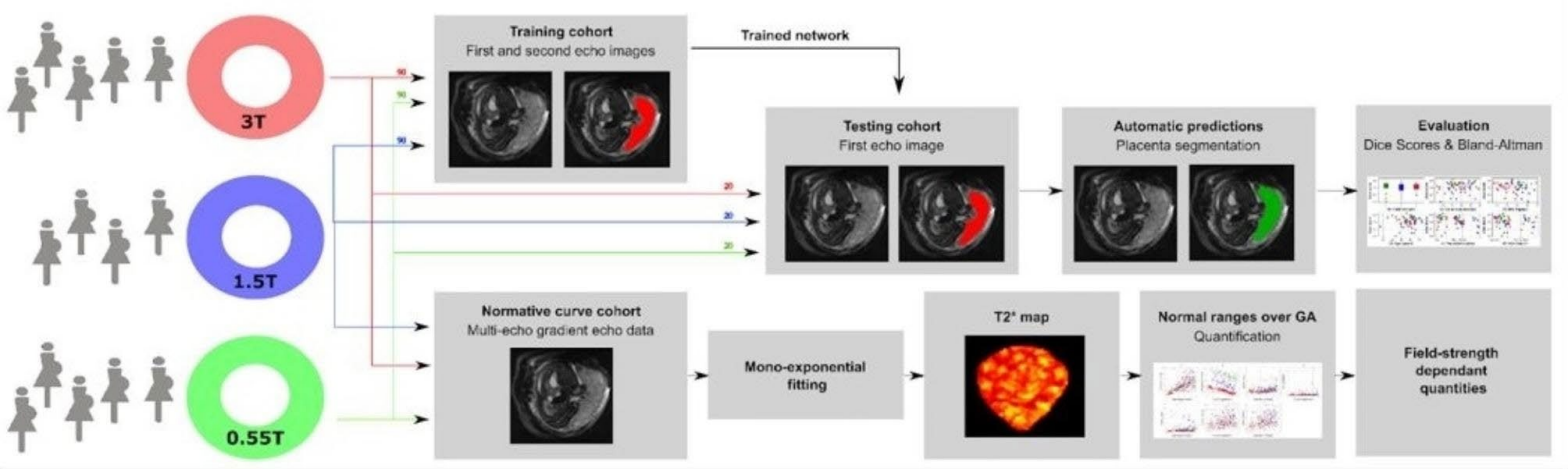
Experimental setup for the cross-field study from the data acquisition (left) to the processing performed including training the network till evaluation of the network results and normative curves.

All protocols commenced with a localizer and T2 weighted turbo spin echo sequences of the entire uterus in sagittal, coronal and axial planes to the maternal habitus. For 3T, a B0 map was acquired and image-based shimming was performed; for 1.5T automatic whole FOV shimming was performed. This step was not required at 0.55T. Next, a multi-echo gradient echo single-shot echo-planar-sequence (MEGE) was acquired in coronal orientation to the mother covering the entire uterus. Resolution varied slightly between 2.5 mm isotropic (1.5T), 3 mm isotropic (3T) and 3.1 mm isotropic (0.55T). Four echo times were obtained at each field strength with the echo times varying slightly from the read-out train length on each scanner (3T: 13.8/70.4/127.0/183.6 ms; 1.5T: 7.84/60.574/113.308/116.041 ms; 0.55T: 46/120/194/268 ms) (Fig. 1).

## Quantification

Conventional scanner vendor reconstruction was performed for this study. No motion correction was performed. Mono-exponential fitting was performed using least-square optimization employing all available echo times (TEs) in python^25^ to obtain T2* maps for the entire imaging volume.

Several quantitative measures were derived, and the mean T2* over the entire organ was calculated. For the placenta a measure of kurtosis and skewness of all voxel values in the resulting T2* maps were calculated. Measures of placental morphology were assessed, as, all other things being equal, these should be field-strength independent, but are expected to change over gestation, and therefore provide helpful validation of data acquisition and analysis techniques. The placental volume was calculated by multiplying the number of voxels in the binary placental masks drawn on the T2* maps by the voxel dimensions. Placental non-uniformity corresponds to a value assigned to every ‘placental’ voxel equivalent to the distance from that particular voxel to the nearest ‘non-placental’ voxel and thus represents the length of the shortest path from each voxel to the edge of the placenta in millimetres. This results in a ‘morphological map’ and metric called placental non-uniformity, calculated by summing the mean and standard deviation of all voxel values. Finally, maximum placental thickness is obtained by multiplying the maximum value in the morphological map by two.

## Automatic placenta segmentation

An automatic placental segmentation was developed using the nn-UNet framework^26^, an adaptive semantic segmentation approach designed to dynamically tailor a segmentation pipeline based on the characteristics of a provided dataset. Through an automated process, it configures a U-Net-based segmentation pipeline specifically adapted to the provided training cases. A full resolution 3D nn-UNet network was trained on a NVIDIA RTX A5000 (fold = 1, batch size = 2, loss = Robust Cross entropy (CE) and Memory Efficient Soft Dice (DS) loss, optimizer = Stochastic Gradient Descent (SGD), epochs: 1000 (250 iterations/epoch), kernel size = [3,3,3] and default nn-UNet augmentation) using both first and second echo of the multi-echo gradient-echo EPI datasets. The training dataset consisted of 210 cases, 70 on each field strength, with its corresponding placental manual segmentations, split 80/20 for training and validation respectively. For these training cases, the placental parenchyma was carefully manually segmented on all slices of the first echo time (TE), avoiding the maternal vasculature (in particular the aortic bifurcation and common iliac vessels often noted when assessing a posterior placenta) and the amniotic fluid by one of three experts (3–9 years of experience in fetal MRI). In addition, a total of 60 cases, 20 on each field strength, were manually segmented by the experts for an independent test set to allow evaluation using calculating the Dice score.

## Normative curves and correlation with outcome

Retrospectively selected and well characterised women who were recruited as healthy controls into research studies were analysed with the automatic segmentation tool and quantification to produce normal curves for all field strengths, assess correlation with gestational age and three key outcome measures. The applied criteria were: 1gestation at birth > = 37 weeks, birth weight centile calculated using INTERGROWTH-21^27^ above the 3rd centile, with exclusions including maternal disease (such as pre-eclampsia or diabetes) at any stage of pregnancy, fetal anomalies or growth restriction diagnosed at any stage of pregnancy, or need for neonatal medical interventions.

### Statistical analysis

All statistical analyses were performed using statsmodels (v0.13.2) and Jupyter Notebook, python3. An ANOVA was used to test for differences in gestational age at scan, maternal age, BMI, placental T2*, T2* z-score, volume, skewness, kurtosis, maximal thickness and uniformity between field-strengths. Benjamini and Hochberg false discovery rate (FDR) was applied to correct for multiple comparisons (reported as p_FDR_). P_FDR_-values < 0.05 were considered statistically significant. Tukey’s Honestly Significant Difference (HSD) test was used to test post-hoc for differences between each field-strength when results from the ANOVA were significant, with these p-values corrected for the family-wise error rate (reported as p_FWE_). Spearman’s correlation coefficient was used to determine the direction and significance of any association between placental T2*, volume, maximal thickness and uniformity and GA at scan for each field strength, and between mean placental T2* z-scores and maternal BMI, maternal age at scan, placental location, gestation at birth or birth weight centile.

## Results

**Fig. 2:**
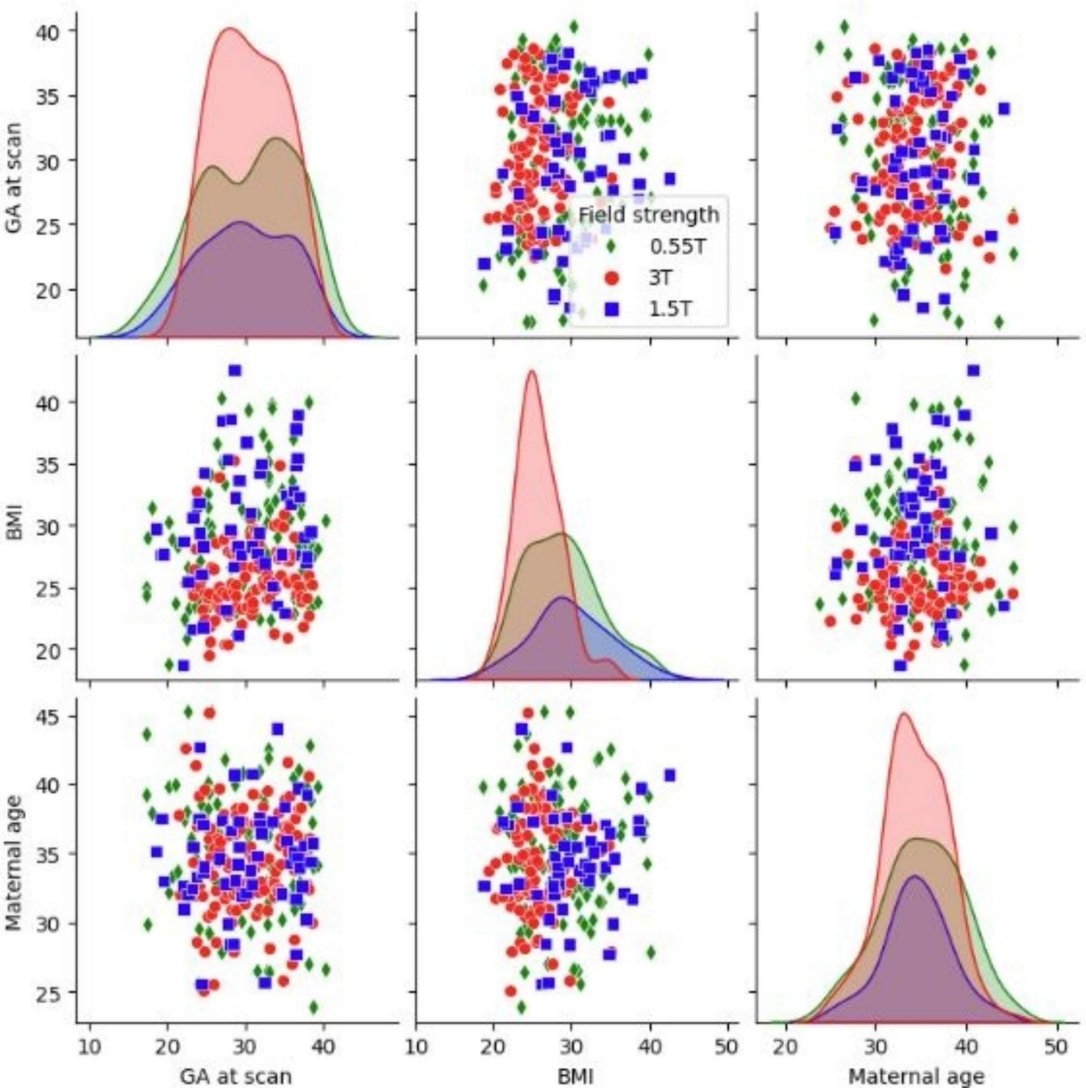
Demographics across all scanners with the cohort on 0.55T in green, at 1.5T in blue and at 3T in red.

**Fig. 3:**
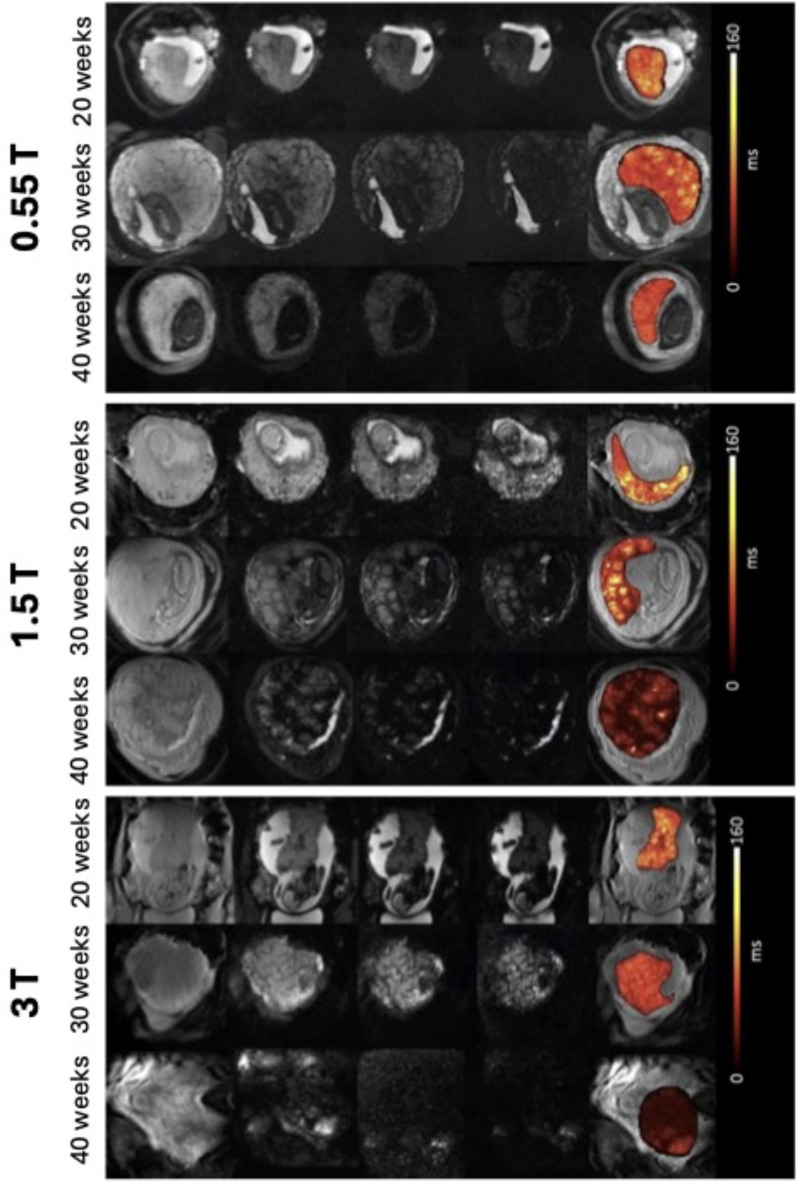
Multi-echo gradient echo data and resulting T2* maps for 20, 30 and 40 weeks gestational respectively at all three field strengths. Scaling differs between field strengths but is consistent within field strength.

A total of 270 scans were included to train and test the automatic segmentation and a total of 273 scans were included into the normal curve evaluation (see Demographics in Table 1; Fig. 2). T2* values could be obtained from all cases. Respective examples across the field strengths for 20, 30 and 40 weeks gestational age are illustrated in Fig. 3 illustrating a visible decrease in T2* at each field as well as the increase in heterogeneity across the placental parenchyma. Geometric distortion artefacts in the form of tissue stretching close to the bowel are visible at 3T.

**Table 1:** Demographics across all cohorts.

	0.55T (normal curves)	1.5T (normal curves)	3T (normal curves)	Training Segmentation 70@0.55T, 70@1.5T, 70@3T	Test Segmentation 20@0.55T, 20@1.5T, 20@3T
N (scans)	98	55	120	210	60
GA [weeks]	30.29+-6.04 [17.43, 40.28]	29.89+-5.53 [18.58, 38.57]	30.34+-4.43 [15.72, 38.57]	28.22+-5.58 [16.0, 40.28]	28.57+-5.45 [17.85, 39.28]
BMI [kg/m2]	28.69+-4.78 [18.72, 40.22]	29.94+-5.01 [18.75, 42.59]	25.72+-2.97 [19.44, 35.19]	28.00+-5.29 [18.75, 49.82]	29.24+-4.62 [21.48, 39.27]
Maternal age [years]	35.04+-4.53 [23.84, 45.21]	34.50+-3.74 [25.52, 44.03]	34.33+-3.60 [25.01, 45.13]	35.17+-5.14 [18.80, 48.73]	34.09+-4.07 [18.35, 40.39]
GA@birth [weeks]	39.66+-1.27 [37.0, 42.86]	40.02+-1.27 [37.14, 42.14]	40.02+-1.20 [37.14, 42.43]	37.91+-4.09 [20.13, 42.14]	39.23+-2.04 [30.42, 42.14]
Birth weight centile	59.63+-28.75 [4.71, 99.95]	71.31+-21.76 [16.96, 97.50]	56.83+-26.20 [4.62, 99.29]	59.36+-29.21 [0.02, 98.43]	52.47+-30.17 [4.86, 99.01]

## Segmentation

**Fig. 4:**
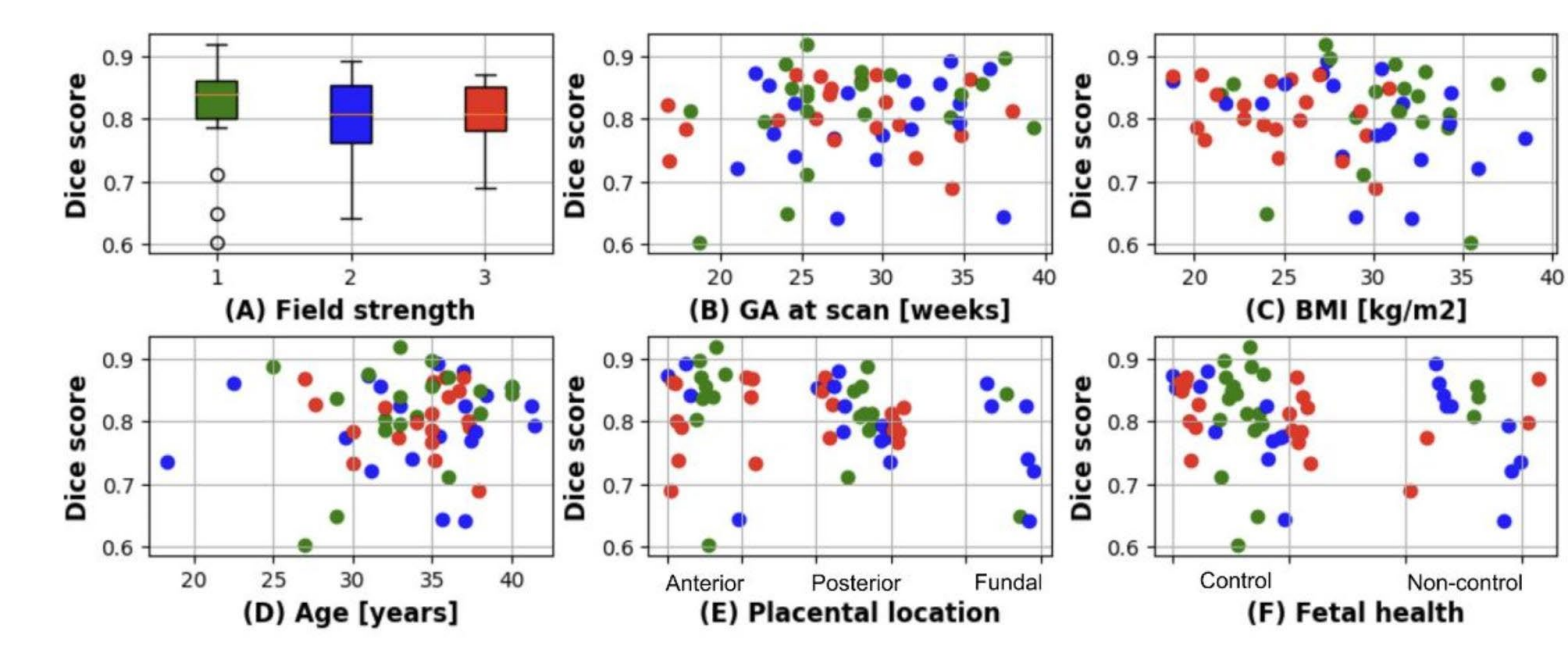
Dice scores for the automatic segmentation results shown (**A**) as boxplots for all three field strengths and in (**B**-**F**) against GA at scan, BMI, Maternal age, placental location and fetal health. 0.55T: green; 1.5T: blue, 3T: red.

**Fig. 5:**
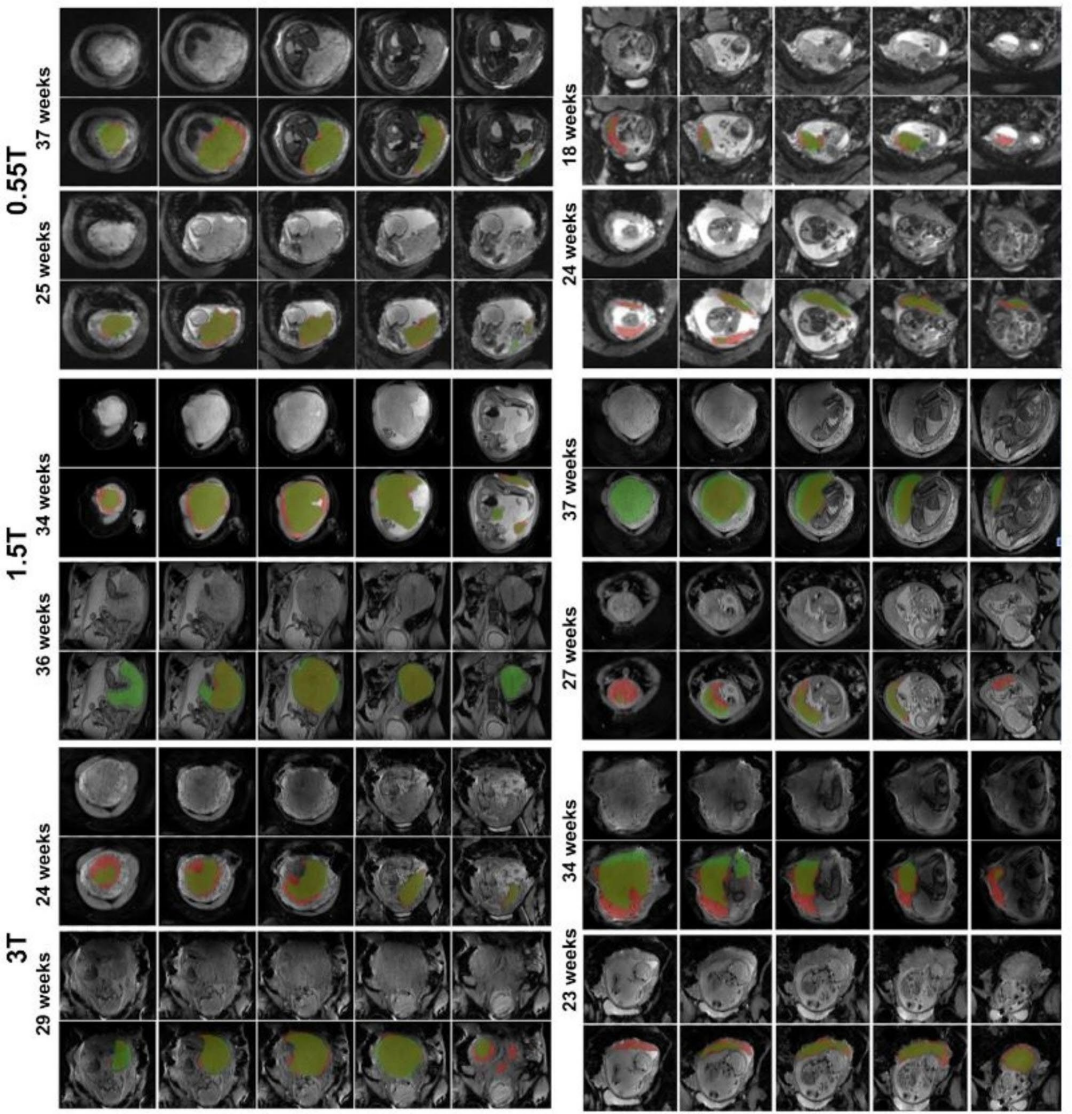
Overlapped manual (red) and nn-UNet predicted (green) segmentation results for the two highest cases (left column) and two lowest (right column) cases at (top) 0.55T, (middle) 1.5T and (bottom) 3T.

The deep-learning placenta segmentation framework was successfully trained on 210 scans (70 from each field strength). The trained nn-UNet model achieved a mean dice score of 0.807 (range [0.69, 0.87]) at 3T, 0.796 (range [0.63, 0.89]) at 1.5T and 0.815 (range [0.60,0.91]) at 0.55T. Network performance was evaluated against GA at scan, BMI, maternal age, placental location and fetal health and showed no significant correlation with any of the parameters as shown in Fig. 4. Graphical examples from cases with the two lowest and highest dice scores for each field strength are shown in Fig. 5.

### Normative curves

Results are summarised in Table 2. The demographics (Table 1; Fig. 2) show that there was no significant difference in mean maternal age (F = 1.06, p_FDR_=0.35) or gestational age at scan (F = 0.34, p_FDR_=0.71) between field strengths. However, mean BMI was significantly different between field strengths (F = 22.0, p_FDR_<0.001). Post-hoc analyses showed mean BMI was significantly lower at 3T (25.72 kg m^− 2^) than at 0.55T (28.69 kg m^− 2^, p_FWE_<0.001) and at 1.5T (29.94 kg m^− 2^, p_FWE_<0.001), but not significantly different between 0.55T and 1.5T (p_FWE_=0.20).

**Table 2:** Summary of statistical results.

Normal Values	0.55T (*N* = 98)	1.5T (*N* = 55)	3T (*N* = 121)
Correlation to GA at scan			
• Mean T2* [ms]	⍴ = -0.747 *p* < 0.0001 R^2^ = 0.56	⍴ = -0.720 *p* < 0.0001 R^2^ = 0.52	⍴ = -0.798 *p* < 0.0001 R^2^ = 0.64
• Placental volume [ml]	⍴ = 0.58 *p* < 0.0001 R^2^ = 0.33	⍴ = 0.61 *p* < 0.0001 R^2^ = 0.38	⍴ = 0.43 *p* < 0.0001 R^2^ = 0.18
• Placental kurtosis [au]	⍴ = 0.60 *p* < 0.0001 R^2^ = 0.36	⍴ = 0.53 *p* < 0.0001 R^2^ = 0.28	⍴ = 0.45 *p* < 0.0001 R^2^ = 0.20
• Placental skewness [au]	⍴ = 0.70 *p* < 0.0001 R^2^ = 0.49	⍴ = 0.55 *p* < 0.0001 R^2^ = 0.30	⍴ = 0.18 *p* = 0.06 R^2^ = 0.03
• Placental uniformity [au]	⍴ = 0.16 *p* = 0.12 R^2^ = 0.03	⍴ = 0.35 *p* = 0.012 R^2^ = 0.12	⍴ = 0.25 *p* = 0.0085 R^2^ = 0.06
• Placental thickness [au]	⍴ = 0.30 *p* = 0.0029 R^2^ = 0.09	⍴ = 0.34 *p* = 0.013 R^2^ = 0.12	⍴ = 0.28 *p* = 0.0035 R^2^ = 0.08
Correlation z-score mean T2* to confounder			
• Maternal BMI	⍴ = 0.051 *p* = 0.63 R^2^ = 0.0026	⍴ = 0.018 *p* = 0.90 R^2^ = 0.0003	⍴ = -0.068 *p* = 0.48 R^2^ = 0.0047
• Maternal age at scan	⍴ = -0.025 *p* = 0.19 R^2^ = 0.0006	⍴ = 0.13 *p* = 0.34 R^2^ = 0.018	⍴ = -0.001 *p* = 0.99 R^2^ = 0.0001
• Placental location	⍴ = 0.14 *p* = 0.19 R^2^ = 0.019	⍴ = -0.11 *p* = 0.42 R^2^ = 0.013	⍴ = -1.01 *p* = 0.30 R^2^ = 0.010
Correlation z-score mean T2* to confounder			
• GA at birth [weeks]	⍴ = -0.00066 *p* = 0.95 R^2^ = 0.0000	⍴ = 0.54 *p* = 0.0088 R^2^ = 0.30	⍴ = 0.35 *p* = 0.002 R^2^ = 0.12
• Birth weight centile	⍴ = 0.22 *p* = 0.037 R^2^ = 0.046	⍴ = 0.062 *p* = 0.78 R^2^ = 0.0039	⍴ = 0.021 *p* = 0.065 R^2^ = 0.046

**Fig. 6:**
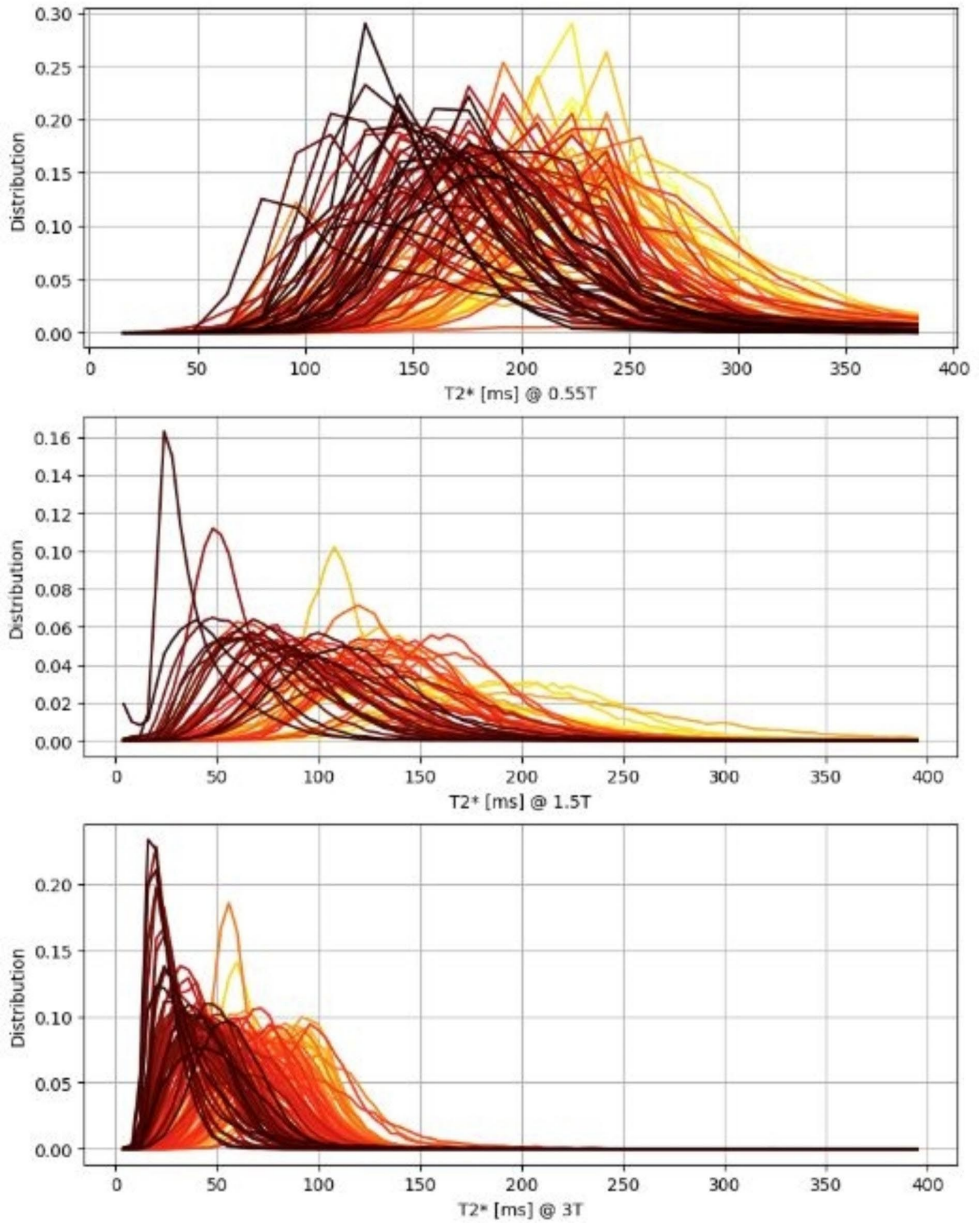
Histograms of placental T2* voxel values for all three cohorts, coloured by gestational age at scan (bright yellow @ 16 weeks to dark red @ 40 weeks).

Histograms demonstrating placental T2* voxel values across the three field strengths are shown in Fig. 6, coloured by gestational age and illustrating visually the left shift trend and increasing kurtosis and - for 1.5T and 3T - skewness.

Placental T2* showed a significant negative correlation with GA at scan at all field strengths (0.55T: ⍴=-0.75, *p* < 0.001; 1.5T: ⍴=-0.72, *p* < 0.001; 3T: ⍴=-0.80, *p* < 0.001), and was significantly different between field strengths (F = 583, p_FDR_<0.001). Post-hoc analyses showed placental T2* was significantly higher 0.55T than 1.5T (p_FWE_<0.001) and 3T (p_FWE_<0.001), and higher at 1.5T than 3T (p_FWE_<0.001) (Table 3).

**Table 3:** Predicted normal values for mean placental T2* across gestation at all three field strengths.

Field strength	20 weeks	30 weeks	40 weeks
0.55T	245 [238–252] ms	200 [196–204] ms	152 [146–159] ms
1.5T	160 [148–171] ms	110 [105–116] ms	70 [57–82] ms
3T	98 [92–101] ms	60 [58–62] ms	25 [21–29] ms

Placental T2* kurtosis was not significantly different between field strengths (F = 1.92, p_FDR_=0.18).

Placental T2* skewness was significantly different between field strengths (F = 6.96, p_FDR_=0.003). Post-hoc analyses showed placental T2* skewness was significantly higher 3T than 0.55T (p_FWE_<0.001), but that there was no significant difference between 3T and 1.5T (p_FWE_=0.12) or between 1.5T and 0.55T (p_FWE_=0.54).

**Fig. 7:**
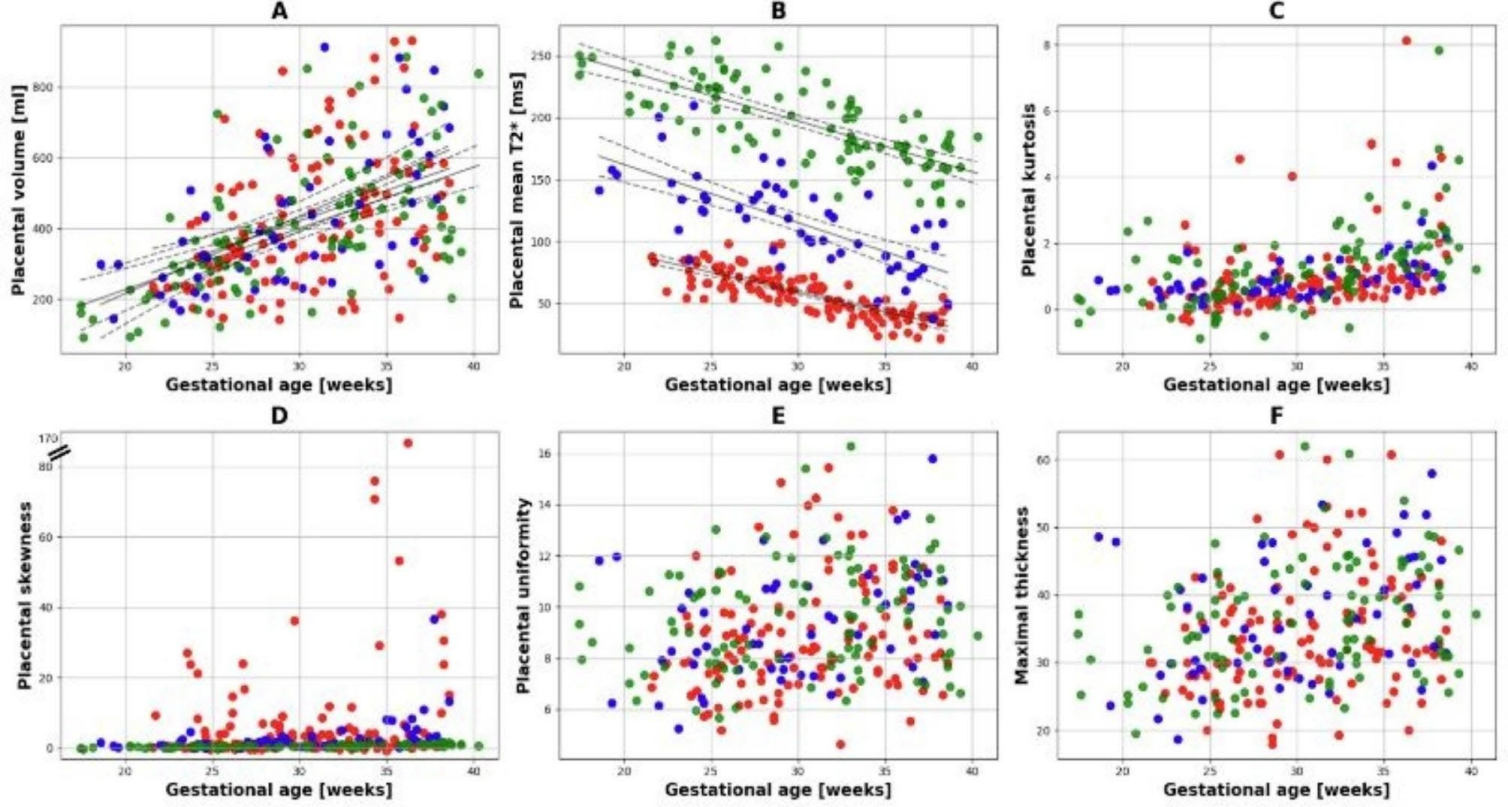
Placental analysis: (**A**) placental volume, (**B**) placental mean T2*, (**C**) placental kurtosis, (**D**)skewness, (**E**) placental uniformity, and (**F**) maximal thickness over gestation for all three field strengths.0.55T: green; 1.5T: blue, 3T: red.

There was no significant difference in placental volume, maximal placental thickness or placental morphology between field strengths (All p_FDR_>0.058). However, these measures all showed a significant positive correlation with GA at scan: placental volume (⍴=0.54, p_FDR_<0.001), maximal placental thickness (⍴=0.31, p_FDR_<0.001) and placental morphology (⍴=0.24, p_FDR_<0.001) (Fig. 7).

**Fig. 8:**
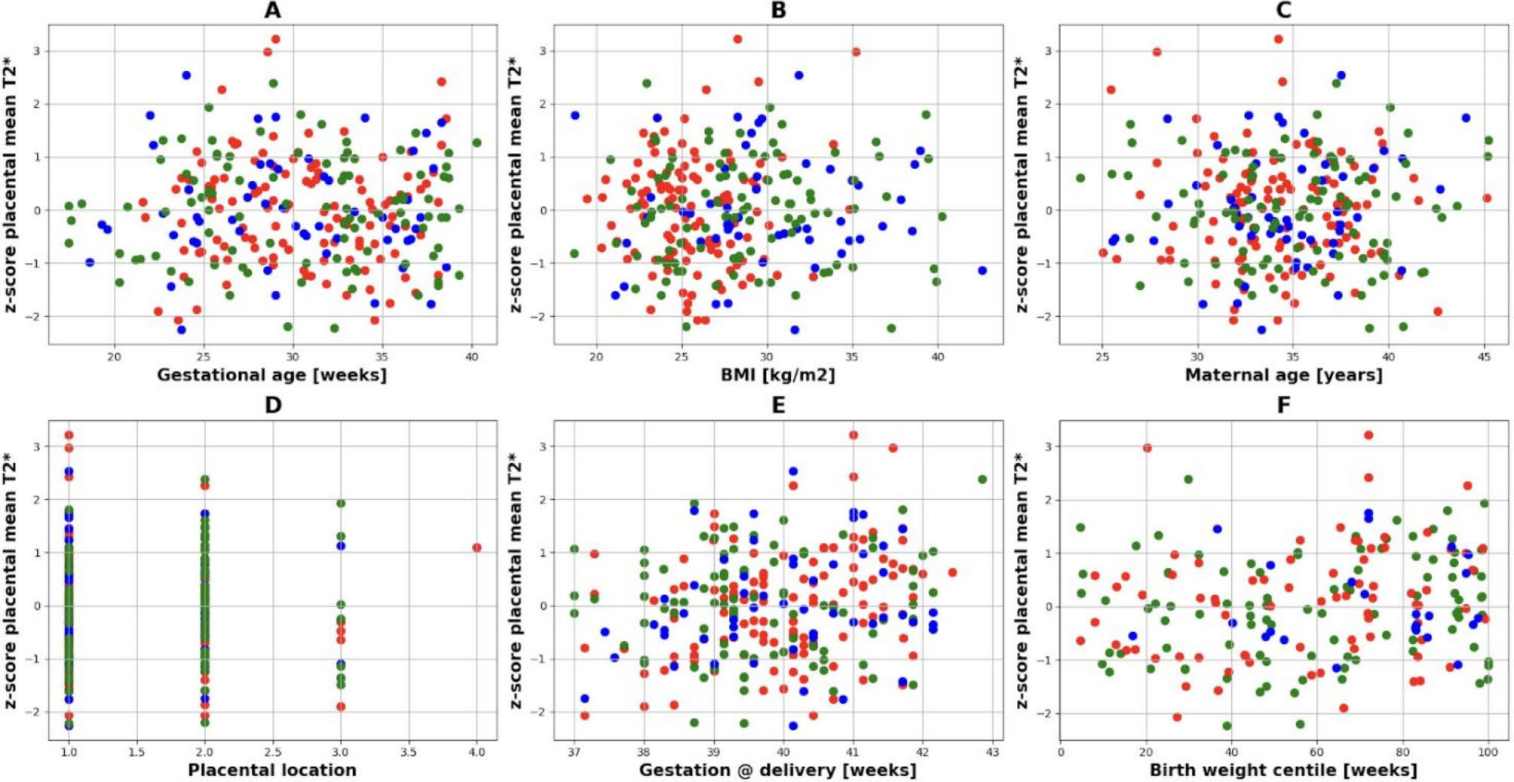
Z-score values are shown against gestational age (**A**) as well as against possible confounders, (**B**) BMI, (**C**) maternal age and (**D**) placental location. Gestation at delivery (**E**) and birth weight centile (**F**) as outcome measures. 0.55T: green; 1.5T: blue, 3T: red.

There was no significant difference in mean placental T2* z-scores between field strengths (F = 0.03, p_FDR_=0.97). There was no significant association between mean placental T2* z-scores and gestational age at scan (⍴=-0.021, p_FDR_=0.76, Fig. 8A), maternal BMI (⍴=0.024, p_FDR_=0.74, Fig. 8B), maternal age at scan (⍴=-0.00033, p_FDR_=0.99, Fig. 8C) or placental location (⍴=0.0081, p_FDR_=0.91, Fig. 8D) (Fig. 8).

There was a significant association between placental T2* z-scores and gestational age at birth in two of the three cohorts(⍴=0.54, p_FDR_=0.0088 at 1.5T and ⍴=0.35 and p_FDR_=0.002 at 3T, Fig. 8E) and birth weight centile in the third (⍴=0.22, p_FDR_=0.037 at 0.55T, Fig. 8F).

## Discussion

This study presents fully automatic quantification of placental T2* MR imaging across all MR field strengths used for fetal MRI. A cross-field network for automatic placenta segmentation was trained and is complemented with several quantitative measures - providing the first comparative placental T2* MRI assessment across field strengths and allowing comparison of normal placental T2* varies across different field strengths. We demonstrate similar changes in placental T2* values, uniformity, thickness and histogram values across all field strengths. No correlation with s BMI, maternal age and placental location was detected at any field strength.

Automatic segmentation of the placenta in MRI is key for accurate and efficient analysis, especially when dealing with large datasets. This is even more important for quantitative techniques, relying on analysing data acquired at different contrast settings together. However, the constraints of the applied contrast setting often reduced the quality of these data sets and thus influences on the required segmentation. Examples are single-shot gradient echo EPI for T2* fitting or diffusion-weighted single-shot spin echo EPI for ADC quantification, both with a resolution typically lower than anatomical T2-weighted data sets and quickly changing contrast between neighbouring tissues. Recent publications on such quantitative data, e.g., by Abulnaga et al.^16^ and Pietsch et al.^17^ explore automatic placenta segmentation in BOLD images, with a cohort limited to one field strength and not taking into account factors like maternal position or gestational age.

The inverse relationship between T2* and field strength is well established, and has been demonstrated in other human tissues: in the adult brain mean regional and whole brain changes in a small cohort has been demonstrated^28,29^; similar results have been shown in musculoskeletal tissues at clinical and high-field strengths. This study demonstrates an inverse relationship between T2* and field strength which is in line with previous studies. However, the data also clearly illustrates, that the longer T2* values at low field are particularly beneficial for placental assessment, where the larger dynamic range may allow for finer discrimination in late gestation. Furthermore, while we have focused on establishing analysis and ranges for normal placentas, T2* was also shown to be reduced in cases affected e.g., by placental insufficiency, similarly benefiting from techniques with a larger dynamic range. The possibility for finer grained analysis, e.g. of histogram shapes was demonstrated. Clear differences regarding the artefacts are also observed between the results at different field strengths. While the data at 3T is affected by geometric distortion artefacts despite image based shimming, the data at lower field strength shows no such artefacts; nonetheless, while placental volume can be determined relative to gestational age using an EPI sequence, the use of a T2 sequence is likely to give a truer placental volume owing to reduced geometric distortion of the sequence over an EPI sequence at all field strengths.

We have utilised large datasets across three field strengths, all of which confer some clinical or research advantage. Acquisition protocols were robust and optimised for all field strengths, and regions of interest were conservatively segmented - both manually for the training dataset and therefore also by the network - to prevent inaccurate mean T2* values either by incorrectly identified regions of interest or partial volume effects. Analysis of multiple parameters, including those that were anticipated to be field strength independent, improved the reliability of our results and provides confidence that measurements such as volume, uniformity and thickness can be extrapolated across datasets. The calculation of normal ranges, and as a consequence the z-scores, allows clinicians and researchers to combine data or utilise the field strength most advantageous to their work, while being able to utilise prior research pertaining to their area of interest. Examples of processed T2* maps from all field strengths are made publicly available, and the trained network is available on github (ANONYMIZED LINK).

There are some limitations to this work: firstly, the acquisition protocols vary in terms of resolution, acceleration and field of view - adapted to the individual constraints given by the field strength and/or population. The population varies according to the scanner - the mean BMI at low field was larger due to the wider bore size available. No paired experiments (in terms of the same pregnant woman having scans on all three field strengths on the same day) were included due to the considerable demand this would pose to the women. Next, scanners from two manufacturers were employed, increasing generalizability but potentially also resulting in inconsistencies due to different pre-processing steps employed on the scanner before the data are extracted. However, we have maintained the same fitting and analysis routines for all datasets for consistency. In line with most placental T2* work to date no motion correction was performed, as intra-slice motion, from maternal and fetal movement, is effectively frozen due to the multi-echo set up for each slice. Inter-slice motion may affect the data which might result in areas being included twice or not at all depending on the motion pattern, although the implications of this are unlikely to vary by field strength. Segmentations on low field are facilitated by reduced geometric distortions and hence potentially more accurate geometrically, potentially explaining the lower Dice score for 3T data. Small variations in network performance across field strengths may result from field-dependent B0 and B1 inhomogeneities, and the variation in contrast that occurs as absolute T2* values vary across field strength. Finally, the birth weight centile was not available for a significant proportion of the datasets at 1.5T limiting the value of the here conducted statistical analysis for this specific outcome measure at this field strength.

We have demonstrated reliable changes in placental T2* measurements even if imaging is undertaken at a different field strength, but that volumetric or morphological analysis should not be expected to differ between groups. In order to aid future research, we have created standardised scores for T2* across field strengths. In particular, this work gives credence to the ongoing study of fetal MRI at low field, where we demonstrate T2* behaves similarly to higher fields, but where the advantage of the intrinsically longer T2* can be utilised; it should also give confidence in interpretation when lower fields are required for clinical reasons, such as high BMI or late gestation. Creation of z-scores also confers clinical advantage meaning that, while most units are limited to one MRI scanner field strength, they can directly utilise published data from other field strengths; furthermore, automation of placental segmentation reduces expertise required and improves efficiency of obtaining T2* results. Equivalent work determining normal T2* ranges across field strengths for other fetal organs could be undertaken. Furthermore, additional work will focus on including the entire automatic pipeline into the scanner host to allow real-time availability of the z-score for any new placenta during the fetal MRI acquisition. Integration of further clinically relevant techniques, such as diffusion imaging, may add greater granularity to our understanding of placental changes across gestation in both normally progressing and complex pregnancies.

## Data Availability

The data that support the finding of the study are not publicly available due to privacy reasons but are available from the corresponding author (megan.hall@kcl.ac.uk) upon reasonable request.
